# Retained sex toys: an increasing and possibly preventable medical condition

**DOI:** 10.1007/s00384-018-3125-4

**Published:** 2018-07-20

**Authors:** Martin Dahlberg, Martin Nordberg, Emil Pieniowski, Lennart Boström, Gabriel Sandblom, Åsa Hallqvist-Everhov

**Affiliations:** Department of Clinical Science and Education and Department of Surgery, Södersjukhuset, Karolinska Institutet, Stockholm South General Hospital (Södersjukhuset), Sjukhusbacken 10, 118 83 Stockholm, Sweden

**Keywords:** Endoscopic treatment, Rectal foreign body, Gastrointestinal surgery, General surgery, Complication

## Abstract

**Purpose:**

Retained foreign rectal objects may require surgical removal. To estimate the magnitude of this problem, we report the incidence and treatment of retained rectal objects at a large emergency hospital, and calculate incidence rates at the national level in Sweden.

**Methods:**

All local patient records during 2009–2017 with the diagnosis foreign body in anus and rectum (ICD-10 T185) were accessed and analyzed retrospectively. All Swedish in- and outpatient visits during 2005–2016 with the code T185 were accessed from the National Patient Register.

**Results:**

We show an increasing incidence in rectal foreign bodies in Swedish national data. The increase was most noticeable in men, and in our local register there was an overrepresentation of sex toys leading to laparotomy and stoma.

**Conclusions:**

To mitigate surgical cost and comorbidity, policies to decrease the risk of retained sex toys could be considered.

## Introduction

Retained foreign rectal objects is a cause of emergency hospital admission and may require surgical removal. A review described 193 patients 1950–2009, mostly from case reports, where 23% required laparotomy [1]. The only large, register-based study (648 patients from Japan) reported that laparotomy was required in 15% of patients [2], whereas hospital-based case series including at least 50 patients have reported 2 [3], 4 [4], and 9% [5]. The aim of this study was to explore the incidence and treatment of retained rectal objects at a large emergency hospital, and to calculate incidence rates at the national level.

## Methods

Stockholm South General Hospital is a public general hospital, serving a population of 600,000. The emergency department has 130,000 annual visits by patients 15 years and older. We investigated all in- and outpatient charts with a diagnosis of foreign body in the rectum (ICD-10 T185) from Jan 1, 2009 to Dec 31, 2017. Our inclusion criteria were that the foreign body (1) was inserted (not ingested), and (2) was presumed in situ at presentation.

The national incidence was based on diagnostic code (ICD-10) T185 from all outpatient visits and admissions of patients 15 years and older recorded in the National Patient Register [6] and demographic data for the Swedish population [7] for 2005–2016. Surgical procedure codes in conjunction with diagnosis of T185 were also investigated. The regional ethics committee approved the study (Dnr 2017-1176-31). R (version 3.2.4, R Foundation for Statistical Computing, Vienna, Austria) was used for statistical analyses.

## Results

### Hospital-based cohort

Between 2009 and 2017, 85 patients presented with a retained rectal object. Median age was 41 years (range 15–92) and 65 (76%) were males. The majority of incidents were self-inflicted (*n* = 61, 72%). Three cases of sexual abuse were reported. The objects were sex toys (dildos and butt plugs) in 41% of cases. The other 59% consisted mostly of cans, bottles, candles, and eatables. We admitted 63 patients (74%) where bedside retrieval was unsuccessful. In 3 patients, the object spontaneously ejected while awaiting surgery. In general or spinal anesthesia, the object was extracted manually or by the help of instruments (forceps, vacuum-extraction, catheters) in 49 cases, and by colonoscopy in 3. For 8 patients, a laparotomy was required, with extraction either by “milking” the object transanally (*n* = 4), or through a colotomy (*n* = 3). In 1 case, the foreign body was retrieved manually, but the patient developed peritonitis due to a rectal perforation requiring diversion (Hartmann’s procedure). Two patients needed a stoma due to rectal injury. In 1 case, extraction failed, but the foreign body was expelled spontaneously 5 days later. All laparotomies were in men, 6 of whom had retained sex toys. The absolute risk of laparotomy was 17% with sex toys and 4% with other objects, with odds ratio 4.9 (CI 0.8–52, *p* = 0.060 with Fisher’s exact test).

### National incidence

**Fig. 1:**
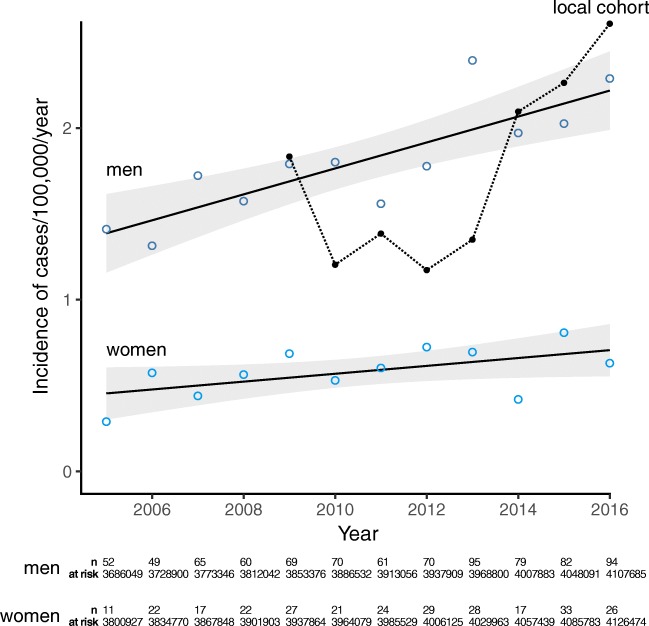
Incidence of retained rectal foreign bodies in Sweden and at Stockholm South General Hospital. National (circles, with linear fit) and local (dotted line) incidence per 100,000 inhabitants/year in inhabitants aged 15 years or older. Aggregate (male and female) incidence is shown for the local cohort

The national incidence rate of diagnostic listing T185 increased from 1.4 to 2.3 per 100,000 person-years between 2005 and 2016 in men (linear fit + 0.08/year, *p* < 0.001) and from 0.3 to 0.6 per 100,000 person-years in women (linear fit + 0.02/year, *p* = 0.054) (Fig. 1). The surgical procedure code sigmoid colostomy was used in 11 patients, and loop ileostomy in 4 in the national data from 2005 through 2016, averaging at 1.3 stomas per year due to retained rectal objects in the Swedish population.

## Discussion

In the present study, 40% of retained foreign rectal objects in a large Stockholm hospital requiring removal under anesthesia were sex toys. Six out of 8 cases needing laparotomy were due to sex toys. National data show that the diagnosis retained foreign rectal body has increased over time.

We hypothesize that a safety string or adequate-sized stopper potentially could have prevented retaining the dildos, since a recurring problem was difficulty in grasping the objects endoluminally. Whereas electronic sex toys are regulated in the EU through the Directive 2011/65/EU of the European Parliament, and general restrictions exist on toxic chemicals in consumer products, other aspects (e.g., risk of retainment) are not covered by any regulations.

Scientific reports on sexual device-related injuries are scarce, but were investigated in the USA based on representative sampling of patients in the National Electronic Injury Surveillance System (NEISS). According to that study, the national incidence rate of sex device-related injuries was 0.24 per 100,000 in 1995 and 0.55/100,000 in 2006, of which 78% were anorectal injuries [8], numbers far lower than ours.

It is difficult to estimate the “true” incidence of sex toy retainment in the population, given reluctance to seek care for a potentially embarrassing condition, and due to insufficient data on exposure. Based on this study, as well as previous data, we believe that retained sex toy is a partly preventable and increasing medical problem, where regulations could impose standardization and enable endoscopic or manual extraction, should the need arise.
